# Botulinum Neurotoxin Induces Neurotoxic Microglia Mediated by Exogenous Inflammatory Responses

**DOI:** 10.1002/advs.202305326

**Published:** 2024-02-11

**Authors:** Ghuncha Ambrin, You Jung Kang, Khanh Van Do, Charles Lee, Bal Ram Singh, Hansang Cho

**Affiliations:** ^1^ School of Medicine University of California San Diego CA 92093 USA; ^2^ Department of Mechanical Engineering and Engineering Sciences University of North Carolina Charlotte NC 28223 USA; ^3^ Institute Quantum Biophysics Sungkyunkwan University, 2066 Seobu‐ro, Jangan‐gu Suwon Gyeonggi 16419 Republic of Korea; ^4^ Department of Biophysics Sungkyunkwan University, 2066 Seobu‐ro, Jangan‐gu Suwon Gyeonggi 16419 Republic of Korea; ^5^ Department of Intelligent Precision Healthcare Convergence Sungkyunkwan University, 2066 Seobu‐ro, Jangan‐gu Suwon Gyeonggi 16419 Republic of Korea; ^6^ Botulinum Research Center, Institute of Advanced Sciences Dartmouth MA 02747 USA

**Keywords:** astrogliosis, Botulinum neurotoxin, complement proteins, human tri‐culture, microfluidics, neurodegeneration, neuroinflammation, proinflammatory microglia, synaptic loss

## Abstract

Botulinum neurotoxin serotype A (BoNT/A) is widely used in therapeutics and cosmetics. The effects of multi‐dosed BoNT/A treatment are well documented on the peripheral nervous system (PNS), but much less is known on the central nervous system (CNS). Here, the mechanism of multi‐dosed BoNT/A leading to CNS neurodegeneration is explored by using the 3D human neuron‐glia model. BoNT/A treatment reduces acetylcholine, triggers astrocytic transforming growth factor beta, and upregulates C1q, C3, and C5 expression, inducing microglial proinflammation. The disintegration of the neuronal microtubules is escorted by microglial nitric oxide, interleukin 1β, tumor necrosis factor α, and interleukin 8. The microglial proinflammation eventually causes synaptic impairment, phosphorylated tau (pTau) aggregation, and the loss of the BoNT/A‐treated neurons. Taking a more holistic approach, the model will allow to assess therapeutics for the CNS neurodegeneration under the prolonged use of BoNT/A.

## Graphical abstract

Microfluidic based humanized mini brain model is utilized to investigate the intoxication with BoNT/A at the CNS, mimicking clinical dosage treatments. Thsese results identifies astrocytes as the key regulator of complement proteins responding to reduced acetylcholine with BoNT/A treatment. Initial neuroinflammation by reactive astrocytes inducing microgliosis. The exacerbated response of the proinflammatory microglia results in synaptic impairment, oxidative stress and neurodegeneration.

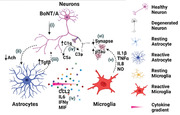

## 1 Introduction

Botulinum Neurotoxin (BoNT) causing botulinum, is synthesized by anaerobic, spore‐forming, gram‐positive bacteria *Clostridium botulinum*. The most interesting aspect of botulinum research is the reformation of the most potent toxin into a protein of significant therapeutic use.^[^ ^1^, ^2^ ^]^ It is considered a therapeutic drug for the treatment of many neurological disorders, such as blepharospasm, strabismus, and even migraine headaches.^[^ ^3^, ^4^, ^5^, ^6^ ^]^ Commercial formulations of isolated toxin (T: incobotulinum, Xeomin) and Complex (C: abobotlinum, Dyspport and onabotulinum, Botox) are the two major forms in which BoNT/A is administered for therapeutics. Isolated toxin (T) is composed of two functional domains, heavy chain (HC) and Light chain (LC) connected by the disulfide bond. The Complex (C) consists of the neurotoxin‐associated protein (NAP's) encapsulating the isolated toxin.^[^ ^5^ ^]^

There are two pathways followed by the toxin at the synaptic terminal. First, at the peripheral nervous system, BoNT/A is localized at the neuromuscular junction as its site of action. The toxin is internalized into the cytosol by binding to the Synaptic vesicle protein 2 (SV2) receptors forming an endosome, followed by its translocation into the cytosol where its proteolytic activity cleaves SNAP‐25 to block neurotransmitter acetylcholine (Ach) release, resulting in flaccid muscle paralysis.^[^ ^7^, ^8^ ^]^ Second, its distal effects have been observed in the CNS presumably by retrograde transportation and transcytosis, interacting with the host cell in a more complex manner than what earlier studies indicated.^[^ ^9^ ^]^ The toxin is internalized into the cytosol by binding to the Synaptic vesicle protein 2 (SV2) receptors forming an endosome, followed by its translocation into the cytosol where its proteolytic activity cleaves SNAP‐25 to block neurotransmitter release, resulting in flaccid muscle paralysis.^[^ ^7^, ^8^ ^]^ Second, its distal effects have been observed in the CNS presumably by retrograde transportation and transcytosis, interacting with the host cell in a more complex manner than what earlier studies indicated.^[^ ^9^ ^]^ In this process, the toxin is endocytosed by SV2‐independent receptors and retrogradely travels to the soma by microtubule‐dependent axonal transport, where it is sorted and released from the primary uptake neuron into the synaptic sites, entering the secondary neurons, while maintaining its enzymatic property.^[^ ^10^ ^]^ Its presence is observed by cleaved SNAP‐25.

in distal neuronal regions. This conclusion has been made based on an experimental determination using both in vivo and in vitro platforms (animal models and microfluidic devices, respectively)^[^ ^9^, ^10^, ^11^, ^12^, ^13^, ^14^ ^]^ at concentrations of the toxin ranging from clinically relevant 100 pm, 10 nm or 30 nm for the ease of experimental determination in most of their experiments. All these studies have demonstrated the concept of retrograde trafficking of the toxin at clinical or subclinical doses. For example, Bomba‐Warczk et al. (2016) demonstrated distal effects at clinically relevant concentrations (picomolar) of BoNT/A but that required an extended incubation period of up to 16 days, which made it impractical to run all the experiments in laboratory conditions. Thus, to reduce the incubation time, they utilized higher concentrations of the toxins for statistical significance that is an important criterion for determining the validity of the experimental observations. Similarly, the amount of Botox used in mice by Caleo et al. (2018) for demonstrating retrograde trafficking was (10 U/kg) within the toxin's clinical dose range. Notably, they detected cleavage of SNAP‐25 specifically in the efferent facial nerve nuclei (FN) in the brain stem that increased between days 3 and 15. Furthermore, Gary Borodic, MD. PhD., an Assistant Clinical Professor in Ophthalmology at Harvard Medical School implies trafficking of the toxin to the central nerves while observing his patents^[^ ^15^ ^]^ and states in his patent, “Botulinum toxin and the treatment of primary disorders of mood”, that “Effects on the central nervous system are observed even when botulinum toxin is administered to the scalp, facial or neck regions, including administration by any form of injection except intracranial injection.”

The major concern when using BoNT is the unintended migration of toxin causing systemic toxicity along its path. Changes in brain activation patterns were observed in clinical patients even after 4 weeks of BoNT/A injections underlying short‐term and long‐term effects of BoNT therapy due to retrograde transportation.^[^ ^16^ ^]^ Abnormal spontaneous brain activity in the CNS was observed in patients with botulism, which could indicate distal effects of BoNT, a phenomenon that has the potential for understanding the adverse effects of BoNT/A as well as its therapeutic opportunities.^[^ ^17^ ^]^ However, it is critical to ascertain the molecular effects of BoNT/A at the cellular level, once it reaches the CNS, as the intracellular enzymatic activity of a single toxin molecule can proteolyze many SNAP‐25 target molecules, providing a considerable amplifying effect.^[^ ^10^ ^]^

In the CNS, the dysregulation in complement proteins (C1‐C9), mainly synthesized by the neurons and the glial cells, are found in pathological conditions, including Alzheimer's disease, Amyotrophic lateral sclerosis (ALS), and Huntington's disease.^[^ ^18^, ^19^, ^20^, ^21^, ^22^ ^]^ In normal conditions, selective elimination of unsuitable synapses takes place by the expression of C1q protein in a classical cascade,^[^ ^23^ ^]^ initiated by the blockage of Ach release. Co‐localization and binding of the C1q to nonfunctional parts, dead cells, and debris results in the autocatalytic downstream complement protein C3. Opsonization of C3 fragments (C3a and iC3b) leads to debris or cell elimination by phagocytosis or lysis by the membrane attack complex (MAC).^[^ ^24^ ^]^ Therefore, C1q and C3 have emerged as critical mediators of synaptic refinement by C3‐dependent phagocytosis of synapses by microglial cells, but what initiates the regulation of C1q secretion remains to be determined. Particularly, TGFβ secreted by astrocytes has been implicated as a crucial regulator for the initiation of the C1q expression^[^ ^25^ ^]^ for synaptic pruning in plasticity. It is termed as having a dual immunoregulatory role^[^ ^26^, ^27^ ^]^ depending on the cell type and conditions governed by cellular context and cytokines. Despite the evidence showing the participation of glial cells in synaptic maintenance through the complementary system and the crosstalk between pathways, the underlying mechanisms are still not clear.

In this study, we performed an investigative assay of BoNT/A by using an engineered 3D human tri‐culture system with human neural progenitor cells in a microfluidic platform.^[^ ^28^, ^29^ ^]^ The 3D construct was formed utilizing human NPC^[^ ^30^, ^31^ ^]^ differentiating into neurons and astrocytes in the center chamber of the microfluidic device. BoNT/A was added to the system after 3 weeks of differentiation in the central chamber. Microglia cells were added after the completion of the BoNT/A treatment, in the outer chamber and monitored for its activation and migration along the microchannels connecting the outer chamber to the central chamber. We investigated the effects of BoNT/A treatment on the brain, layer by layer in a dose‐dependent manner. To this end, our human tri‐culture model enabled us to investigate the immunomodulatory effects of BoNT/A. We observed an increase in microglial engagement with periodic BoNT/A treatment. We also discovered a de novo induced astrocyte response mediated by the loss of acetylcholine. Our model mirrored key representative features of neurodegeneration: activation of complement C1q, C3, NO release, increase in proinflammatory cytokine, pTau aggregation, and synaptic impairment.

## 2 Results

### 2.1 Construction of a Triculture 3D Human Tri‐Culture System to Investigate Neuroinflammation Driven by BoNT/A

**Figure 1:**
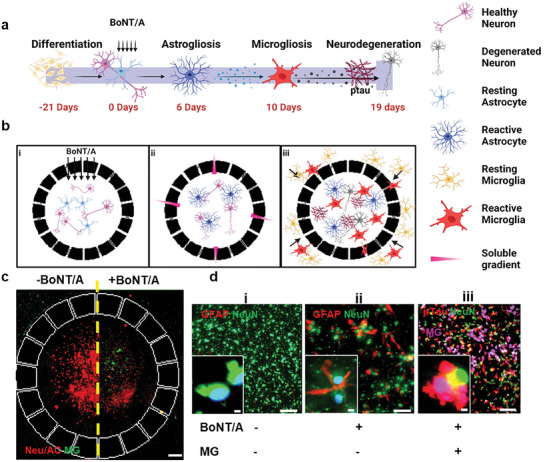
Multi‐dosed BoNT/A treatment, the risk factor for CNS neurodegeneration a) The timeline shows how to reconstitute BoNT/A‐derived CNS neurodegeneration. b) The schematic describes neuron‐glia interactions induced by BoNT/A in an engineered 3D human tri‐culture system. i) Neurons and astrocytes are co‐cultured in a 3D central compartment under repeated exposure to BoNT/A. ii) Intoxicated neurons and reactive astrocytes release cytokines for microglia in an annular compartment. iii) Engaged proinflammatory microglia exacerbate neurodegeneration. C) Fluorescent images compare engaged microglia (green) and reduced neurons and astrocytes (red) with BoNT/A treatment (right) compared to no treatment (left). D) Fluorescent images represent i) untreated healthy neurons (NeuN) and resting astrocytes, ii) intoxicated neurons and reactive astrocytes (GFAP) after five times treatment of BoNT/A, iii) microglia (pink) recruited and colocalized on pTau aggregates (red). Scale bars, c) 500 µm, d) 100 and 10 µm insets .

**Figure 2:**
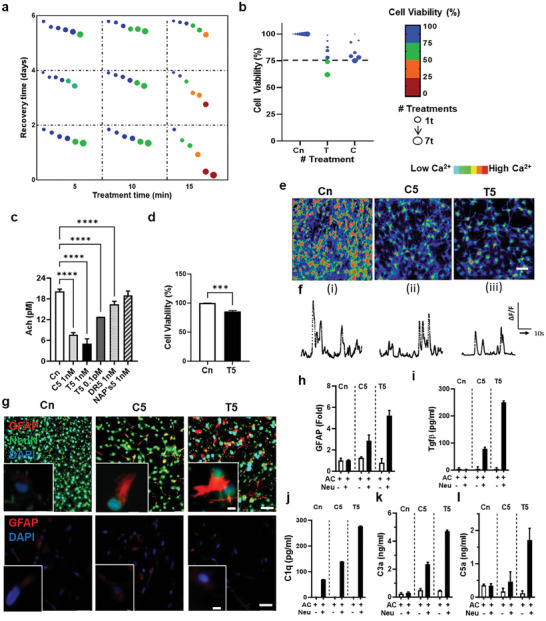
Intoxication of neurons and release of complementary proteins under BoNT/A treatment a) The map provides the thresholds of 1 nm BoNT/A treatment with the variation of treatment time and recovery time (n>3) b) Neuronal viability after five times treatment of T/C forms at 1 nm for 10 min exposure and 2‐day recovery (n>3) c) Reduced secretion of acetylcholine (Ach) from neurons after the BoNT/A treatment (n = 4) experiment sets, repeated twice. d) Significant reduction in cell viability with 0.1pM T5 BoNT/A treatment. e) Ca^2+^ imaging visualizes the reduced electrophysiological activity after the BoNT/A treatment. f) Normalized values (ΔF/F) from neurons representative of control i) Cn, ii) C5, and, iii) T5 treatment, indicating inhibition of calcium signaling, all experiments were repeated ≥3 times. g) Fluorescent images indicate reactive astrocytes (GFAP) in co‐cultured with neurons (upper) and single‐cultured (lower) conditions, respectively, astrocytes gain reactivity in co‐culture with BoNT/A‐treated neurons and, h) GFAP increased expression in the co‐culture model with BoNT/A treatment i–l) Graphs show discernable release of TGFβ, C1q, and C3a in co‐culture, further by T5 than C5. n) Significant expression of C5a by T5 but not as significant by C5 compared to T5. Quantification was done with n = 3 individual wells, in triplicate, and repeated ≥3 times. All parameters are presented as mean ± SD. All of the data were quantified using one‐way ANOVA followed by Sidak's multiple comparison test. ****p <0.0001, ^***^ *p* <0.001, ^**^ *p* <0.01, ^*^ *p* <0.1. Scale bars, h) 50 µm, i) 100 µm and 10 µm inset.

In this study, we set our hypothesis that the decrease in the Ach neurotransmitter release due to BoNT/A treatment (T, C), may induce overexpression of TGFβ from the astrocytes upregulating C1q in neurons, initiating the complement cascade. Once the neuronal cells underwent a complete set of treatments (a total of 5; T5 and C5), the microglial cells were introduced into the system. The microglial cells are activated by the soluble cues generated by the BoNT/A treatment of neurons and astrocytes, resulting in migration and exacerbated neuroinflammatory response. The phosphorylated tau (pTau) leads to the disruption of microtubule organization resulting in synaptic impairment and neurodegeneration (**Figures** 1a and 2e).

To test our hypothesis, we employed our in vitro model in microfluidics tri‐cultured with neurons, astrocytes, and microglia to investigate any intoxication driven by BoNT/A in the CNS. The platform consists of two chambers: the central and the annular chamber connected by the migration channels forming a gradient of soluble factors (Figure 1b,c). The Neuron progenitor cells (NPCs) were added to the central chamber and allowed to differentiate for 3 weeks into neurons and astrocytes creating a physiologically relevant 3D human tri‐culture model. Once fully differentiated, the cells were periodically exposed to BoNT/A in both isolated toxin (T) and complete complex (C) forms. (Figure 1b‐i). Upon the addition of BoNT/A into our models, we observed a significant increase of GFAP in astrocytes, indicating induction of reactive astrocytes by the BoNT/A treatment (Figure 1b‐ii,d‐ii), compared to the control (Fig 1di). Since BoNT/A is known to reduce the secretion of neurotransmitter Ach, we attributed the decreased Ach as a cause of the increased reactivity. The microglial cells were then loaded in the annular chamber, after the completion of the BoNT/A treatment. We observed microglial migration to the central chamber in response to the soluble cues released by neurons and reactive astrocytes in the central chamber (Figure 1b‐iii). Immunofluorescence staining with CD86 demonstrated the induction of proinflammatory microglia by the BoNT/A treatment. Interestingly, most of the CD86‐positive microglia were co‐localized in the regions aggregated with pTau (Fig 1diii). In addition, the microglial cells were co‐localized with the truncated parts of synapses presumably due to the pruning process of damaged neurons mediated by microglia (Figure 1c). Overall, our model validated key aspects of neurodegeneration driven by gliosis under the BoNT/A treatment, such as reactive astrocytes, and colocalization of proinflammatory microglia.

### 2.2 Assessment of BoNT/A Intoxication in Neurons and Astrocytes

To create an environment consistent with the therapeutic dose of administered BoNT/A, the cells (coculture of both neurons and astrocytes) were exposed to the toxin with different exposure and recovery times while maintaining the cell viability >75% (Figure 2a). We observed a decrease in cell viability, reducing it to ≈74% after the 5th treatment with isolated toxin (T5) (Figure 2b). The cell viability was reduced to 78% after the 5th treatment of C5. To maintain the cell viability at >75%, 5th treatment was selected as the last and final treatment, after which the viability decreased dramatically.

Soluble N‐ethylmaleimide‐sensitive‐factor attachment receptor (SNARE) complex comprising of synaptobrevin 2, Syntaxin, and synaptosomal‐associated protein (SNAP‐25) partake in the fusion of intracellular vesicles with the plasma membrane leading to the synaptic release of neurotransmitter, acetylcholine (Ach). Therefore, we investigated the inhibition of Ach with BoNT/A (T5) at two different concentrations, 0.1pm, and 1 nm, demonstrating concentration‐dependent reduction of Ach release (Figure 2c) and cell viability (Figure 2d), **Table** **1**.

**Table 1:** Summary of SNAP‐25 cleavage‐induced reduction in Ach and Cell viability with different concentrations of BoNT/A (T5) treatment.

Conc. T5	Ach decrease (%)	Cell death (%)
1nM	68.7± 5.4	25.6 ± 5.0
0.1pM	36.1±0.1	14.6 ± 1.9

Next, we confirmed the presence of cholinergic neurons in the human tri‐culture model (S6)^[^ ^53^ ^]^ and investigated the proteolytic activity of BoNT/A by measuring changes in the level of Ach secretion. We observed a 68.71% ± 5.43 decrease in Ach by T5 and a 62.6%±3.49 decrease by C5, an indication of substantial loss of neurotransmission. (Figure 2c). We also compared the effects of DRBoNT (detoxified recombinant BoNT+NAPs, DR5) and NAP's (Neurotoxin‐associated protein, NAPs5). While only an 18.4% ± 4.08 decrease was observed with DR5, there was no significant decrease with NAP's5.

We investigated the effects of T5 treatment on neurons, and astrocytes and compared them to C5 treatment. We first measured the Ca^2+^influx with the treatment of both T5 and C5. We found that neurons in the control cells exhibited normal calcium influx in response to the action potential, while the calcium influx was discernibly inhibited in the presence of T5 (32.15%±0.855) and C5 (29.17%±0.98) (Figures 2e,f and 4b; Figure S7, Supporting Information).

We next explored the intoxication effects of toxins on astrocytes, as the astrocytes are the major glial cells gaining reactivity through their communication with neurons that are involved in eliminating damaged synapses.^[^ ^29^, ^30^, ^31^, ^32^, ^33^ ^]^ To this end, we employed single‐cultured astrocytes as well as co‐cultured models of neurons and astrocytes, exposed to 1 nm BoNT/A (S2). Afterward, we performed immunostaining of glial fibrillary acidic protein (GFAP) to estimate the level of astrocyte reactivity (Figure 2g,h). Our data showed that astrocytes, only co‐cultured with neurons, were significantly activated by both T5 (sixfold) or C5 (threefold) treatments compared to the non‐treated cells (Figure 2g,h; Figure S2, Supporting Information). However, the single‐cultured astrocytes did not exhibit any reactivity, indicating that astrocytes were not directly impacted by the BoNT/A (S2).

Interestingly, our data showed that astrocytes in the co‐cultured model treated with either T5 or C5 significantly increased the level of both TGFβ and C1q. TGFβ expression by T5 was threefold more than the C5 treatment, while C1q expression was twofold more with the T5 treatment (Figure 2i,j). To further validate the increased astrocyte reactivity in the co‐cultured models under the BoNT/A treatment, we measured the amount of C1q, a major soluble factor released by reactive astrocytes promoting neuroinflammation which has been previously reported (Figure 2j; Figure S4, Supporting Information).

Since the complement cascade generated anaphylatoxins and opsonins, we further estimated the amount of C3a and C5a in the conditioned media, using ELISA (Figure 2k,l). We observed that C3a was significantly increased in co‐cultured models by both T5 treatment (15‐fold) and C5 treatment (sevenfold) compared to the single‐cultured models or controls. We found a 4.8‐fold increase of C5a in our treatment with T5 and only 28% upregulation with C5 treatment. Our results confirmed the activation of C1q followed by the production of C5a and C3a, which may further drive proinflammatory pathways in the human tri‐culture exposed to BoNT/A toxins.

### 2.3 Implication of Astrogliosis in Promoting Cytotoxicity

**Figure 3:**
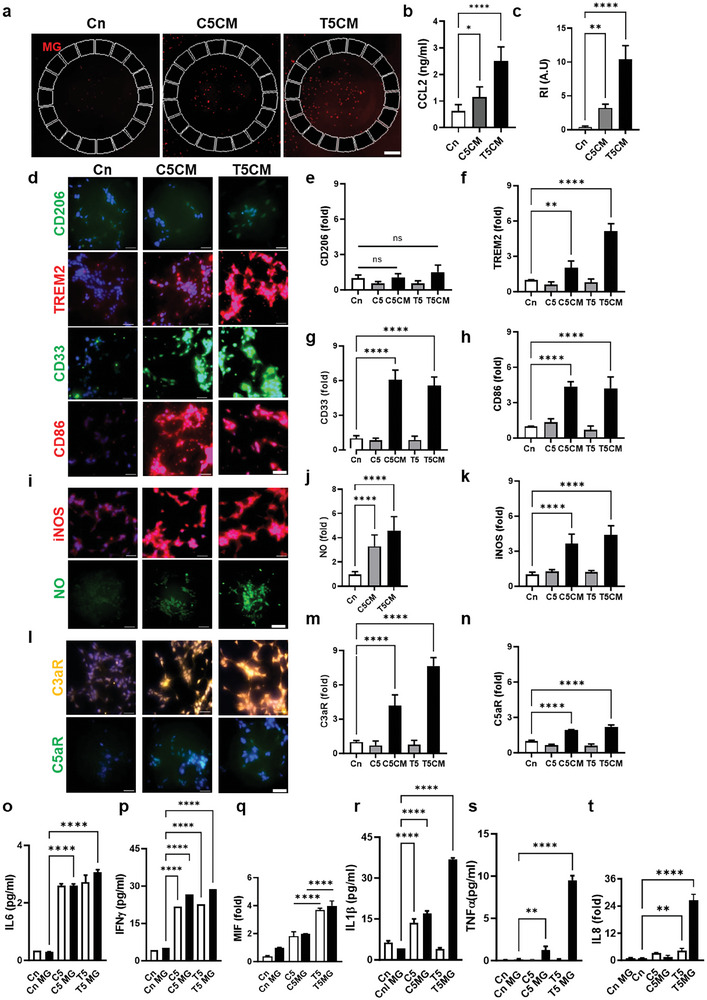
BoNT/A‐induced microglial proinflammation mediated by complementary proteins a) Microglial (red) engagement is confirmed in real‐time in response to T5/C5‐treated neuron‐astrocyte conditioned media (T5CM/C5CM), b) The microglial chemokine, CCL2 was present in the T5CM, more than C5CM. c) Microglial recruitment index (R.I) indicates a significant response to T5CM compared to C5CM, (n = 10). d–h) Immunofluorescent images show the microglial conversion to a proinflammatory phenotype with CD86 but no anti‐inflammation with CD206. Scavenger activities were confirmed with TREM2 and CD33 (n≥ 3). i‐k. The inducible isoform iNOS (n = 3) is overexpressed and microglia consequently produce neurotoxic NO under T5CM treatment more than C5CM (n = 8). l–n) Complement activation was confirmed with C3aR and C5aR on microglia (n >3 sets), driving the microglia into proinflammatory phenotype. m‐o) Inflammatory cytokines, IL6, IFNγ, and MIF compared to the control, were found discernably in T5CM and C5CM but not augmented by microglia. p–r) Inflammatory cytokines, IL1β, TNFα, and IL8 were significantly measured from T5CM‐treated microglia compared to C5CM‐treated microglia (n = 12, all parameters are presented as mean ± SD). All of the bar graphs were quantified using one‐way ANOVA followed by Sidak's multiple comparison test. ^****^ *p* <0.0001, ^***^ *p* <0.001, ^**^ *p* <0.01, ^*^ *p*<0.1 and “ns”, non‐significant. Scale bars, a) 500 µm, d,i.l) 50 µm.

The role of the increased number of cytokines with BoNT/A treatment (Isolated toxin and complete complex) is not well known in the recruitment of microglia. To identify the mechanism of the microglia activation and recruitment, we added the adult microglial cells to the BoNT/A treated coculture of neurons and astrocytes in the 3D microfluidic device (**Figure** **3**a). We observed that the microglial migration toward the central chamber started after 48 h. The microglial recruitment index, R.I. (a measure of the number of cells migrated to the central chamber over 9 days was 10.40 ± 2.04 by the T5CM and 3.19± 0.57 by the C5CM (Figure 3b), suggesting that the complex form of the toxin showed less effect on microglial recruitment. We identified CCL2 (fivefold increase) as the chemoattractant in the T5CM inducing microglial migration and activation (Figure 3c and Table 3). Since CCL2 was a chemoattractant involved in microglial recruitment, the amount of CCL2 was not calculated after the addition of microglia to the system (Table 3).

To determine the response of microglia to the BoNT/A, we treated the human microglial cells directly with the T5 and the C5 and conditioned media (T5CM, C5CM) from the T5 and C5 treated coculture of neurons and astrocytes. We observed the activation of microglia only in T5CM and C5CM (Figure 3; Figure S3, Supporting Information). Once activated, the microglia phenotype can be modified to M1 or M2. In our study the pro‐inflammatory phase is recognized as the M1 phase, which is identified with the overexpression of the marker CD86, CD33, and TREM2 (Table 3), modulating the pathology toward phagocytosis and neurodegeneration (Figure 3d–h). The M2 neuroprotective phase, identified by the CD206 marker, was not expressed (Table 3), suggesting that the phenotype was not neuroprotective (M2 phase). Expression of inducible nitric oxide synthase (iNOS) and NO is activated (Figure 3i–k and Table 3; Figure S3, Supporting Information), implying neuroinflammation.

The immunofluorescence staining of Complement receptors C3aR and C5aR revealed that both receptors were significantly expressed in the treatment conditions (T5CM/C5CM) (Figure 3l–n and Table 3). There was approximately a fourfold increase of C3aR compared to C5aR. The results indicated that the BoNT/A (T5/C5) did not have a direct effect on the microglia as none of the receptors overexpressed upon direct exposure to the toxin. The data also suggested that the effect of BoNT/A was only mediated through the neural cells for glial cell activation. The microglial phenotype changes to M1 state modulating proinflammatory cytokine secretion and oxidative stress, leading to neurodegeneration. The dramatic upregulation of C3aR suggests that the bound C3a activates the microglial receptors thereby triggering elimination by phagocytosis making it the major pathway for the elimination of nonfunctional synapses. Furthermore, C5 complement upregulation contributes to many pathological processes with inflammatory phenotypes.

The CM was further screened for cytokines and we observed that IFNγ, IL1β, IL6, TNFα, MIF, IL8, and IP10 were overexpressed by the microglia in response to BoNT/A treatment in both isolated as well as complete complex forms (Figure 3o‐t and **Table** **2**; Figure S5, Supporting Information). Since the amount of secretion of IL6 (Figure 3o and **Table** **3**) was found to be approximately the same between the CM obtained from the T5/C5 treated co‐culture model and triculture model, we can say that the astrocytes were the key regulators of its secretion.^[^ ^49^ ^]^ Microglia adds (18%−21%) to the secretion of IFNγ (Figure 3p) that is upregulated by the astrocytes in the coculture model (Table 3). MIF is found to be expressed by both the astrocytes and the microglia (Figure 3q and Table 2). The data implicated microglia in the secretion of IL1β, TNFα, and IL8 with T5CM treatment of microglia whereas it did not play a dramatic role in the C5CM treatment condition (Figure 3r–t).

**Table 2:** Summary of the fold increase of cytokines and Chemokines with BoNT/A treatment.

Cytokines	C5 (Fold)	C5 MG (Fold)	T5 (Fold)	T5 MG (Fold)
CCL2	2.10± 0.75	NA	4.68±0.92	NA
IL1β	2.22±0.21	3.08±0.36	0.64±0.04	6.60±0.63
TNFα	1.00±0.03	1.60±0.34	1.02±0.05	8.00±0.46
IL 6	7.94±0.19	7.95±0.17	8.33±0.77	9.39±0.26
IL 8	3.30±0.15	1.70±0.71	4.20±1.09	26.49±2.70
IFNγ	5.44±1.88	6.69±2.20	5.63±1.70	7.14±2.10
MIF	1.80±0.29	1.97±0.05	3.28±0.03	3.99±0.29

**Table 3:** Summary of the fold increase of the microglial receptors treated with BoNT/A (T5/C5) condition media.

Markers	C5CM (Fold)	T5CM (Fold)
CD 206	1.1± 0.3	1.5±0.6
TREM2	1.9±0.4	5.0 ±0.9
CD 33	6.0±0.7	4.0 ±0.7
CD 86	4.3±0.4	4.2 ±0.9
iNOS	3.6±0.8	4.2± 0.8
NO	3.4±0.9	4.7±1.1
C3aR	4.1±0.9	7.6 ±0.7
C5aR	1.9± 0.0	2.2±0.1

### 2.4 Assessment of Neurodegeneration by BoNT/A in a Triculture model

**Figure 4:**
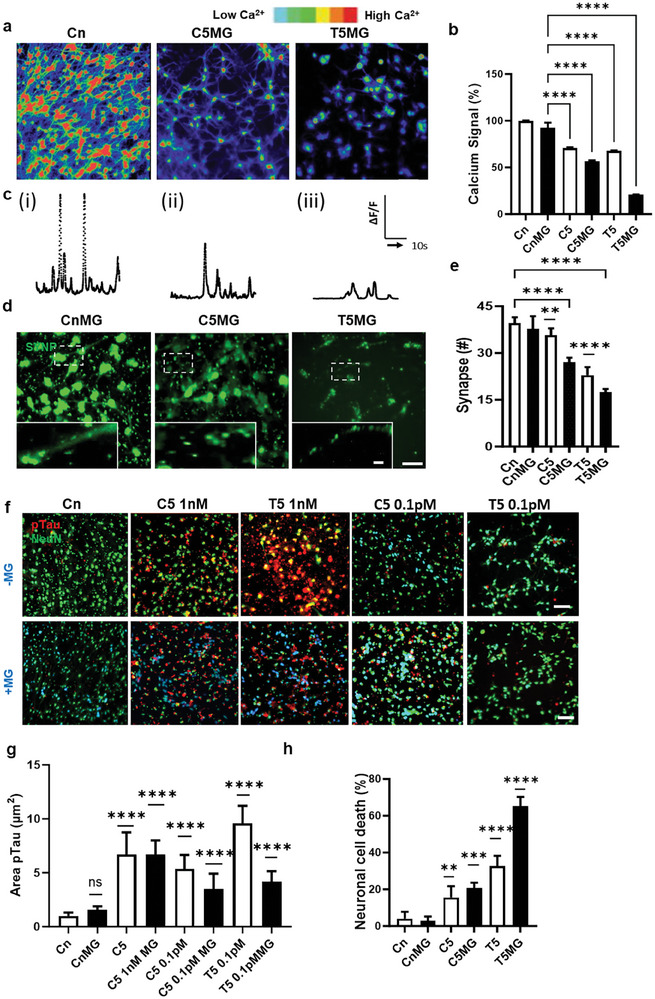
Neurodegeneration exacerbated by BoNT/A‐induced microglial proinflammation a) Ca^2+^ images show further neurodegeneration by BoNT/A‐induced proinflammatory microglia. b) Measured Ca^2+^ signaling indicates electrophysiological activities reduced significantly by T5CM‐treated (T5MG) and intermediately by C5CM‐treated microglia (C5MG). c) Normalized values (ΔF/F) from neurons representative of i) control (Cn), ii) C5 and, iii) T5 treatment. Calcium signaling is further reduced in the presence of microglia as determined by the number and intensity of the peaks. All experiments were repeated ≥3 times. de) Synaptic impairment was assessed by counting immunostained synapses (SYNP). f–h) Neuronal damage was assessed by measuring the sizes of pTau aggregates (red) and the number of neurons (green) after microglial treatment at higher concentrations (1 nm), and lower concentrations (0.1pm) of BoNT/A in both isolated and complex forms. pTau aggregates (red) were significantly increased after the exposure to the toxin T5 and C5 compared to C5 at 1 nm concentration. Severe neurodegeneration was observed under T5CM‐microglial co‐culture compared to C5CM‐microglial coculture. Scale bars, a) 50 µm), d) 50 and 10 µm insets, f) 100 µm. All experiments were repeated ≥3 times; all parameters are presented as mean ± SD. p values were calculated using one‐way ANOVA followed by Sidak's multiple comparison test. ^****^ *p* <0.0001, ^***^ *p* <0.001, ^**^ *p* <0.01, ^*^ *p* <0.1 ^****^ *p* <0.0001, ^***^ *p* <0.001.

Microglial cells were added to the dual culture of neurons and astrocytes after the completion of the 5th treatment with BoNT/A (T5/C5). Ca^2+^ flux imaging of cells in the triculture model revealed a depletion of calcium influx after BoNT/A treatment that was further reduced after the addition of the microglial cells. In the C5‐BoNT/A treatment, the calcium influx was reduced by 29.3 ± 0.98%, further reduced to 56.73 ± 0.88%, with the addition of the microglial cells. With the isolated toxin T5‐BoNT/A treatment, the Ca^2+^ influx reduced to 32.15 ± 0.49% reducing even further to 20.82 ± 0.11% with the addition of microglial cells. The data implies that the synaptic function is significantly reduced by the BoNT/A (T5/C5) treatment that is further impaired by the microglial cells (**Figure** **4**a–c).

We further verified this by immunofluorescent staining with synapsin (SNYP) antibody. With the T5 treatment the number of functional synapses reduced by 43.24 ± 7.65%, which further reduced to 56.4 ± 2.54% with the addition of microglia cells. The number of functional synapses reduced by 17.95 ± 6.34% by the treatment with C5, were significantly further reduced to 30.76% ± 4.3 with the addition of microglia. (Figure 4d,e).

To assess neurodegeneration, the presence of p‐tau phosphorylation was also confirmed by immunofluorescent staining using the p‐tau antibody (Figure 4f–h; Figure S8, Supporting Information). The p‐tau aggregation increased 9.9 ± 0.1‐fold with T5 and 6.6 ± 2.0‐fold with C5 treatment. With the addition of microglia to the BoNT/A treated cells, we found that the microglia cells behaved differently. It made no effort to clear the aggregation in the C5‐treated cells, whereas it reduced the aggregation 3‐folds in the cells treated with T5 (Figure 4f,g) and exacerbated neuronal cell death by 65.2% ± 5.06 (Figure 4h). The neurotoxic effect of the microglia was found even at low concentrations of BoNT/A (0.1pm) with both T5 and C5 treatment conditions (Figure 4g,h).

### 2.5 Identifying C1q as a Risk Factor for Microgliosis in Response to Reduced Ach by BoNT/A

In our model, we included additional evidence demonstrating the complement proteins as the risk factor for initiating the inflammatory response. We have employed DRBoNT/A, a catalytically deactivated version of BoNT/A and NAPs preparation devoid of catalytic ability, to demonstrate that C1q is responsible for the initiation and progression of neuroinflammation in combination with microglial response.

**Figure 5:**
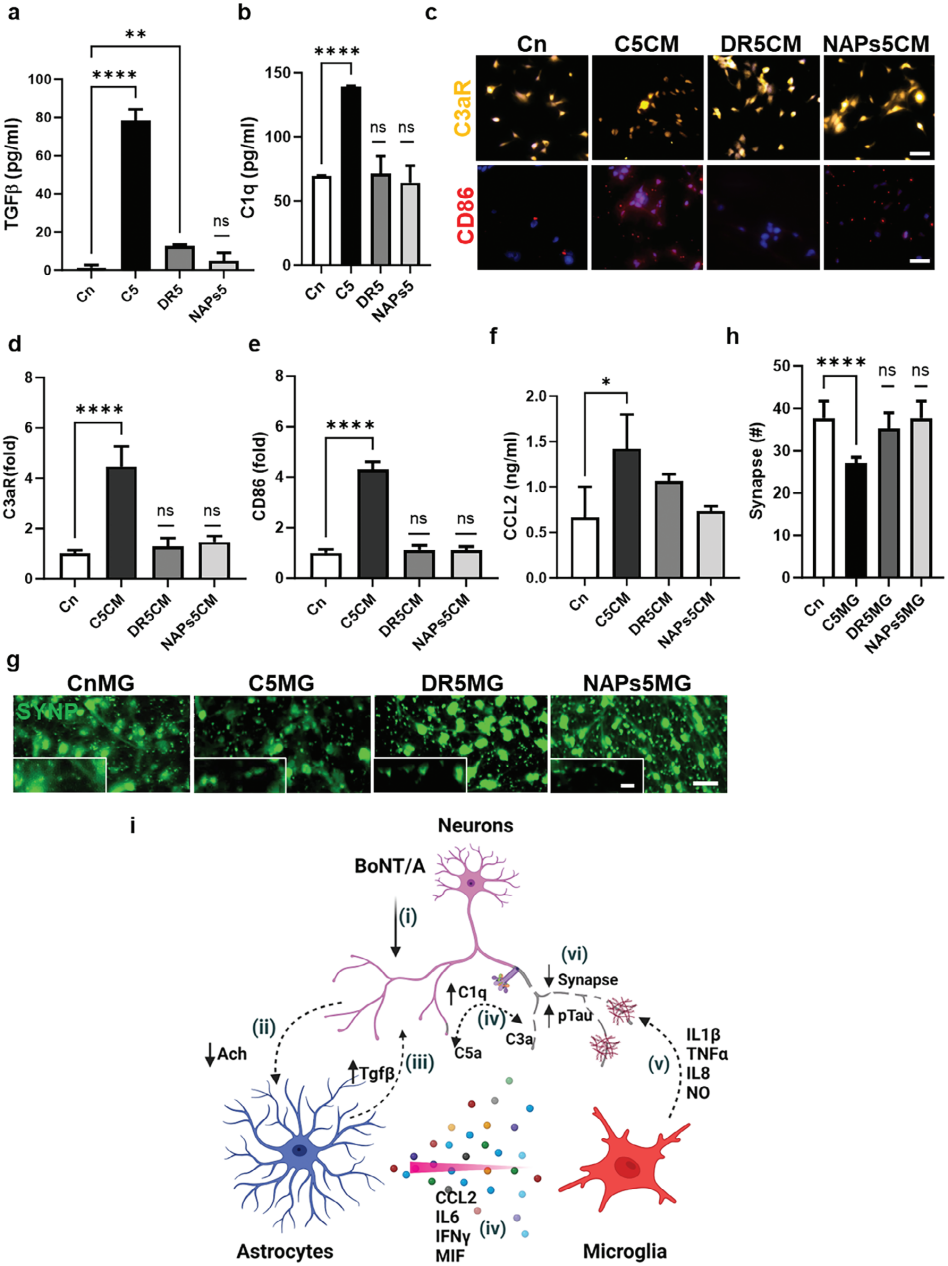
Activation of Complement cascade. a) TGFβ is expressed substantially more in C5 treatment compared to DR5 or NAPs5 treated cells. b) C1q was not significantly expressed with either DR5 or NAP's5 treatment. c–e) Immunofluorescent images show no significant microglial conversion to a proinflammatory phenotype with CD86 and C3aR upon treatment with DR5CM and NAPs5CM, hence not driving the microglia into a proinflammatory phenotype. f) Chemoattractant CCL2 was not significantly expressed with DR5CM and NAPs5CM treatment. g,h) No significant change in the number of synapses was observed with DR5MG and NAPs5MG. i) The schematic summarizes our mechanism study on BoNT/A‐derived CNS neurodegeneration. Scale bar: 50 and 10 µm insets. All experiments were repeated ≥3 times; all parameters are presented as mean ± SD. p values were calculated using one‐way ANOVA followed by Sidak's multiple comparison test. ^****^ *p* <0.0001, ^***^ *p* <0.001, ^*^ *p* <0.1^****^.

We identified TGFβ as the driving force of C1q secretion in agreement with other research groups.^[^ ^25^, ^60^ ^]^ We compared TGFβ expression of DRBoNT (detoxified recombinant BoNT+NAPs, DR5) with C5 as C5 secretion was less than T5 treatment. TGFβ was discernibly less expressed (83.5% ± 1.5) with DR5 treatment compared to C5 treatment (**Figure** **5**a).

We further investigated the expression of C1q with DR5 and NAPs (Neurotoxin‐associated protein, NAPs5) treatments, comparing with the C5 treatment. No Significant expression of complement protein C1q was observed with DR5 and NAP's5 treatment (Figure 5b).

To determine the response of microglia to the DRBoNT/A and NAPs, we treated the human microglial cells with conditioned media (DR5CM and NAPs5CM) from the DR5 and NAPs5 treated coculture of neurons and astrocytes. No significant upregulation of C3aR (Figure 5c,d) suggests that the Complement pathway was not activated.

To determine if microglia phenotype can be modified to the proinflammatory M1 phase assessed by CD86, no significant upregulation of CD86 was observed with DR5CM and NAPs5CM (Figure 5e). Microglia Chemoattractant CCL2 was not significantly upregulated with the DR5CM and NAP's5CM (Figure 5f).

We then investigated microglia‐mediated synaptic pruning by immunofluorescent staining with synapsin and observed no significant reduction in synapse with DR5MG and NAPs5MG (Figure 5g,h).

Complement system has long been implicated in the immune surveillance in the brain, extending beyond inflammation and host defense. It can be produced locally in the brain in response to injury or inflammatory signals by neurons and glial cells. In a healthy human tri‐culture, the complement protein (C1q) is majorly secreted by the astrocytes and microglia. Complement expression by neurons has so far been described mainly in the disease context and upon injury to the CNS.^[^ ^14^, ^15^ ^]^ In our experimental conditions, it is brought about by the BoNT/A treatment in complete complex and isolated toxin forms. The BoNT/A treatment results in the depletion of Ach from the system leading to the activation of the classical pathway mediated by the TGFβ overexpression. The classical pathway is activated when the C1q binds to the defective synapses following a conformational change, leading to the channeling of the C1q molecule to generate C3 and C5 complement proteins. Cleavage of C3 generates anaphylactic peptides C3a and C3b/iC3b, which lead to the elimination of complement‐bound synapses by phagocytosis, mediated by the microglia. In addition to direct mediators of microglial engulfment, signaling by the chemokines and cytokines expressed by the neurons and astrocytes leads to synaptic elimination (Figure 5i). Neuroinflammation including reactive astrocytes, microglial activation, activation of the complement cascade, oxidative stress, and, synaptic impairment are fundamental features of neurodegeneration, which have been observed in our experiments. These observations suggest that the 3D model successfully demonstrated the microglial involvement potentially in the detrimental effects of long‐term BoNT/A treatment on the healthy human tri‐culture system.

## 3 Discussion

Endotoxin is a lipopolysaccharide (LPS) mostly found in the outer membrane of gram‐negative bacteria.^[^ ^33^ ^]^ Botulinum neurotoxin is a gram‐positive bacterium having no outer membrane and hence contains no endotoxin. Gram‐positive bacteria carry surface teichoic acids, lipoteichoic acids (LTA), and peptidoglycan. aLTA is known to promote apoptosis by the NF‐κB pathway, while bLTA does not produce an inflammatory response to induce apoptosis. lipoteichoic acids (LTA) are found only on Clostridium butyricum, the exotoxin of which is Type E.^[^ ^34^, ^35^ ^]^ These contribute to sepsis and multiple organ dysfunction syndrome^[^ ^36^ ^]^ activating the host immune cells to have an immunomodulatory effect. The peptidoglycan of Clostridium botulinum was found to be diaminopimelic acid (DAP). The upregulation of cytokines has not been reported on any DAP‐associated gram‐positive bacteria in the literature. In our study, we have employed botulinum neurotoxin, type A that is an exotoxin of gram‐positive Clostridium Botulinum.

The ability of botulinum neurotoxin to disrupt neurotransmission, for a prolonged period, is exploited for its use in medical applications and the cosmetic industry. It now presents a therapeutic choice for the treatment of several neuromuscular conditions. BoNT has been shown to have effects inside the CNS by blocking at least one neurotransmitter that could be acetylcholine (Ach), glutamate, norepinephrine, etc., each having a specific and vital function in human physiology. BoNT/A cleaves specifically at residues 197–198.^[^ ^37^, ^38^, ^39^, ^40^, ^41^, ^42^, ^43^, ^44^, ^45^ ^]^ Cleavage of SNAP‐25 appeared preferentially in cholinergic terminals despite their relatively low density in the Facial Nucleus (FN) present in the brain stem.^[^ ^14^ ^]^ The IC_50_ of neurotransmitters excluding Ach from cultured neurons in vitro was 0.32± 0.05 mg mL with ≈18 nm BoNT/A after 3 days of treatment,^[^ ^46^ ^]^ whereas complete inhibition of Ach was observed after only 2 h of treatment at 2 nm concentration.^[^ ^47^ ^]^ Moreover, Ach plays a vital role in neuroplasticity within the cortex and hippocampus, and our research could provide ways to utilize the toxin for nerve regeneration in conditions like brain stroke. The system we have developed could also be utilized for examining disorders like Alzheimer's and Parkinson's diseases that are associated with a decrease in Ach, implying the utility of BoNT as a tool to examine molecular mechanisms involved in the neurodegenerative pathways.

With over 50 million treatments every year, several side effects related to CNS neurological diseases have started to emerge.^[^ ^48^ ^]^ Using our newly established 3D human tri‐culture model, it became possible for us to examine the effects of BoNT/A on the CNS, mimicking the treatment of BoNT/A in a dose‐dependent manner in a highly controlled and physiologically relevant microenvironment. Compartmentalization further facilitated the visualization of microglial engagement through the channels in response to the cell‐specific chemical cues, creating a chemotactic gradient, thus allowing for the assessment and quantification of the BoNT/A effect on individual cells used in our system. In this study, we have established that the neurons are the only cell type that is directly affected by the BoNT/A treatment, not the astrocytes or the microglial cells. We tested the proteolytic activity of T5 and C5 against NAP's5 and DRBoNT5, as negative controls for the proteolytic activity. NAP's are the neurotoxin‐associated proteins devoid of the enzymatic part of BoNT/A, and DRBoNT/A is a catalytically deactivated nontoxic version of the BoNT/A.^[^ ^49^ ^]^ We observed no change in the amount of secretion of Ach with the treatment of NAP's as it does not have the machinery to exhibit proteolytic activity (Figure 2c). However, we observed a decrease in Ach secretion with DRBoNT. We hypothesized that this effect could be for two reasons. First, it could be because of the residual effect of BoNT proteolytic activity. Second, there might be other cellular mechanisms involved in facilitating neurotransmission, which is affected by the non‐catalytic domain of the BoNT.

As the physiology of the in vitro model is different from the human model, it is difficult to determine the duration of treatment and the concentration, given the complexity of the human body. 50 nm and higher concentration of BoNT/A has been used in vitro models for determining the endopeptidase activity^[^ ^50^ ^]^ with incubation time, varying up to 96 h.^[^ ^51^ ^]^ To mimic the BoNT/A dosage, we monitored the cell viability as the deciding factor for determining the treatment duration.

Ozawa in his work has implicated the modulatory role of Ach in GFAP expression.^[^ ^52^ ^]^ Therefore, we confer that the depletion of Ach by neurons under BoNT/A influence would contribute to the astrocyte reactivity (Figure 2g,h). We identified the transforming growth factor (TGFβ) as the factor secreted by the astrocytes in response to the decrease in Ach release upon BoNT/A treatment (Figure 2i). TGFβ is the major component responsible for the upregulation of C1q.^[^ ^25^, ^69^, ^70^, ^71^ ^]^ Here we find that both C3 and C5 components are upregulated. The cleavage of C3 and C5 releases the anaphylatoxins C3a and C5a, which function as chemoattractants.^[^ ^53^ ^]^ In addition, activation of astrocytes leads to the secretion of IL6, IFNγ, and MIF^[^ ^54^, ^55^ ^]^ (Figure 3o–q), implicating astrocytes in the progression of neurodegeneration.

This cytokine data in combination with our control experiments of treatment of glial cells with BoNT/A suggests that while the microglia cells were not directly affected by the BoNT/A (T5/C5), their activation, however (Figure 4) was mediated by the enhanced level of cytokines secreted from the coculture of neurons and astrocytes. The higher recruitment of microglia is due to a combination of factors. 1. The activation of the complement cascade (as indicated by increased C1q; Figure 2j–l) results in the downstream activation of C3 fragmentation, which helps opsonize the neuronal cell surface tagging them for phagocytosis.^[^ ^56^ ^]^ 2. An increased expression of the chemokine (C‐C motif) ligand 2 (CCL2), also referred to as monocyte chemoattractant protein 1 (MCP1), triggering the recruitment of the microglial cells (Figure 3b). 3. Expression of Triggering receptor expressed on myeloid cells 2 (TREM2), leading to a cascade of events promoting the engulfment of synaptic proteins (Figures 3d,f and 4d,f). These findings provide novel insights into pathways linking BoNT/A treatment to potential neurodegeneration. Because of its responsiveness to the microenvironment, microglia can be used as diagnostic markers for contributing to the outcome of neurodegenerative diseases.

IFNγ has been recognized as having a paradoxical role in neuroinflammation, nevertheless, it can be recognized as a proinflammatory cytokine driving immune responses. In this study we observed an upregulation of IFNγ in both T5CM as well as C5CM compared to the control, however, there was no significant difference in its expression between these two conditions (Figure 3p). Despite no difference in IFNγ expression, we observed a significant change in the oxidative stress (Figure 3i–k,p) and the response of the microglia (Figure 4f–h) between these two conditions. The overactivation of Complement C3 and C5 (Figures 2k,l and 3l‐n) could be modulating the upregulation of iNOS with concomitant production of NO in T5CM compared to C5CM.

Macrophage migration inhibitory factor (MIF) is a multipotent cytokine involved in promoting proinflammatory response.^[^ ^57^ ^]^ It is known to be the key factor for the activation of microglia in chronic neuroinflammation and possibly accelerates neurodegeneration.^[^ ^56^, ^58^ ^]^ It has also been implicated in controlling the cytokine release related to tau hyperphosphorylation and neurodegeneration.^[^ ^59^ ^]^TGFβ, IFNγ (Figures 2i and 3p), and complement factors^[^ ^60^ ^]^ are known to stimulate the expression of MIF.^[^ ^60^ ^]^ With the information of all the proinflammatory cytokines that are stimulated by the MIF from previous research,^[^ ^57^, ^60^ ^]^ we can safely suggest that the MIF stimulated the release of proinflammatory cytokines TNFα and IL8 from macrophages in the system treated with BoNT/A (Figure 3s,t). MIF is also known to stimulate nitric oxide (Figure 3‐i,k) production that can directly mediate cell injury.^[^ ^61^ ^]^ In our results, we also observed NO to be overexpressed with BoNT treatment in both C5 and T5 conditions. This stimulation for NO production could be directed by MIF or regulated by other cytokine expressions in the BoNT/A treated system, damaging synaptic plasticity. A clear difference in the effects of T5 and C5 treatments will be critical in explaining the difference in the potential to cause neurodegeneration. Further investigation is important to correlate the form of toxin and its potentially damaging effects. If these differences are due to the size^[^ ^50^ ^]^ or structural differences between the two proteins or if NAPs play an additional role in modulating a change in response, and would these differences mediate cytotoxicity, neurodegeneration, or asymptomatic condition, is yet to be elucidated.

Our 3D human tri‐culture system showed a robust accumulation of p‐tau in neurons treated with BoNT/A. Here we observed the most dramatic differences between the two forms of BoNT/A treatment (T5 and C5). The amount of p‐Tau aggregation was drastically amplified with T5 treatment compared to the control (≈tenfold) and that was 30% more than the p‐Tau level after a separate treatment with C5. An intriguing aspect was the involvement of microglia. While we noticed an aggressive removal of p‐tau in the T5MG treated cells (≈fivefold decrease compared to T5), we observed no significant change in p‐Tau clearance between C5MG and C5 treated cells (Figure 4f–h). It is unclear if this is regulated by MIF that is more significantly expressed (1.5‐fold) in T5 condition compared to C5 (Figure 3q) or if Complement C3 modulates the behavior of microglia as we find it to be significantly upregulated (≈twofold) in T5 condition compared to C5 (Figure 2k), thereby enhancing the phagocytic activity of the microglia in the clearance of pTau (Figure 4f,g). Further studies need to be done to elucidate if the condition in C5 treatment is asymptomatic wherein the microglia fail to clear the aggregated pTau despite the biomarkers expressed. Could any other pathways be more active with the treatment of C5, and act to counter microglia engulfing as the BoNT effect is known to modify transcriptional regulation?.^[^ ^55^ ^]^ It is not clear why the microglia chose to engulf the aggregated p‐tau from the T5 treatment and not from the C5 treatment and needs further investigation. The biological system is very complex and this needs to be further studied and assessed to determine if some other factors or pathways initiate the engulfment of the p‐tau aggregation by the microglia that is present in the T5 but is absent in C5.

Synaptic dysfunction and loss are common elements in many human CNS neurodegenerative disorders.^[^ ^62^, ^63^ ^]^ We observed a decrease in synapses that was reduced significantly upon the introduction of microglia (55% with T5 and 30% with C5). This observation confirms our hypothesis of the sequence of events that leads to synaptic elimination by direct removal of the nonfunctional synapses by phagocytic uptake by the microglia. Since there was a significant difference between the isolated toxin and complete complex on factors that would affect microglia activation, likely, a multiplex pathway (e.g., Complement, caspase, etc.^[^ ^65^ ^]^) may be involved in the toxin‐induced neurodegeneration. These findings suggest that once the microglia are overactivated, neuronal damage is amplified, inducing widespread damage to the neighboring neurons.

Neurodegenerative diseases are known to involve a complex interplay between immune signaling, genetics, and neural damage that results in debilitating cognitive impairment. The neuronal and synaptic loss resulting in neurodegeneration can be mediated by the accumulation or aggregation of protein phosphoryl microtubule‐associated protein tau (p‐Tau) in response to neuronal damage, as we see in our model. Together our results prove that microglia may be indirectly involved in neuronal demise mediated by the astrocytes, a distinct possibility of connecting the repeated BoNT/A treatment to tau pathology and synaptic impairment driven by Complement cascade.

Previous evidence has revealed the neuron‐to‐neuron movement of BoNT/A, allowing it to spread within the neuronal network to have distal effects.^[^ ^9^ ^]^ The data described above indicate the detrimental effects of BoNT/A (C and T treatment in a dose‐dependent manner even in low concentrations (0.1 pm; Figure 4f,g) once it reaches the CNS. While we used 1 nm to establish the mechanism of the toxin action in the CNS, we conducted experiments at clinically relevant concentrations (0.1pm). The same pattern of tauopathy was observed at subclinical levels for both the C5 and T5 forms of BoNT/A. (Figure 4g). A 5.3‐fold increase in aggregation was observed with the C5 treatment and a sevenfold increase with T5 treatment. Notably, 0.1 pm, corresponding to ≈2–5 units of BoNT/A therapeutic dose, is the minimally used treatment of neuromuscular disorders. We monitored pTau aggregation by immunocytochemistry, where the neurons were stained with NeuN (green) and pTau Aggregation with pTau (red).

Since the additional proteins associated with BoNT could be responsible for the activation of neuroinflammation we, therefore decided to measure by arrangement on the exclusion of other factors other than enzymatic activity by using DRBONT and NAPs. The Neurotoxin‐associated protein (NAPs) is structurally and genetically significantly similar to BoNT/A and BoNT/B sequence (≈20%) and is always positioned next to the BoNT gene. Despite being similar in structure, it does not have the HExxH zinc‐binding motif characteristic of Clostridial neurotoxin metalloproteases,^[^ ^66^, ^67^ ^]^ hence is not catalytically active. DrBoNT/A is a double‐mutant E224A/E262A full‐length botulinum neurotoxin (BoNT) Type A with structural similarity to native BoNT/A but lacking the endopeptidase activity.^[^ ^68^ ^]^ Since they are structurally similar to the native Toxin and lack proteolytic activity, we decided to use these two compounds to determine if the C1q pathway is activated in the absence of proteolytic activity and if microgliosis is influenced in the absence of C1q.

Since we observed a decrease in Ach with DrBONT/A treatment, we further examined the expression of TGFβ and C1q that is the initial responder of the complement pathway. The expression of TGFβ by DR5 and NAPs5 was significantly less than even C5 that induced no significant secretion of C1q to begin with (see Figure 5a,b). We observed no significant amount of Chemoattractant CCL2, C3aR expression with DR5 and NAPs5 treatment compared to the Control. Interaction between microglia and neurons is significantly less, as demonstrated by reduced synaptic pruning. With this set of data, we can safely suggest that the main source of inflammatory response was mediated by C1q incited by synaptic cleavage of SNAP‐25 and reduction of Ach by the proteolytic activity of BoNT/A.

The study established the involvement of the complement pathway in the mediation of the progression of cytokine expression and MIF being the key player mediating the proinflammatory expression and activation of microglial cells to M1 phenotype. In this study, we have observed that the repeated treatment of BoNT/A can have neurodegenerative potential with its long‐term use in therapeutics and cosmetics. Further studies need to be carried out to determine why certain cytokines and pathways are more distinct in T5 treatment compared to C5. In addition, we need to evaluate if other pathways are also distinctly active that can suppress or neutralize the detrimental effect of the neurotoxin. This could display therapeutic benefits in other neurological diseases, like depression, epilepsy, or Alzheimer's disease.

Here, we developed and validated for the first time a human in vitro model providing long‐term concerns of BoNT/A. The developed assay recapitulates physiological features having the whole integrated system wherein multiple changes in biochemical activity are identified. The assay recognizes an overall set of reactions mimicking the complexity of the human brain. This model provides a robust platform that can identify minute changes when treated with different forms of BoNT/A (C,T, DRBoNT, and NAPs) thereby becoming a handy tool for diagnostic purposes. The system is highly sensitive even at lower concentrations (LOD: 0.1 pM), providing better formulation, better treatment options, and a better regime. It provides a diagnostic tool for studying neurodegeneration with high reproducibility and high throughput.

For most of the experiments in this research, higher concentrations were used for the ease of doing experiments, and for clarity in understanding the whole mechanism, by connecting to the experimental conditions used under in vivo experiments. Moreover, lower concentrations required more careful handling of the samples that could lead to lower accuracy due to the protein sticking to the pipette tips or walls of the tube. Since we were mimicking the clinical pattern of treatment, we used lower concentrations to affirm the effectiveness of the system and to observe if it followed the same pattern as we have stated in the manuscript.

The results from our 3D human tri‐culture model suggest a potentially detrimental role of BoNT/A treatment in the CNS mediated by astrogliosis. Although some adverse clinical symptoms have been reported with therapeutic and cosmetic use of BoNT/A,^[^ ^27^ ^]^ however, it is not clear how long of a treatment it takes to have a clinical effect. No system currently is in place to evaluate the preexisting mental condition of patients to evaluate possible signs of aggravated neuroinflammation upon treatment with BoNT/A. This study provides detailed valuable information on the direct central effects of BoNT, which could be harnessed for its safe use in therapeutics. Further investigation is required to understand the pathway mechanism undertaken in these conditions. Studies involving the blocking of signaling and selectively targeting cytokine effectors would display improved safety by preserving host immune defenses. Moreover, inhibition studies will further ameliorate and present tractable targets for its blockage. These scientific certainties and doubts, addressing future research investigations with inhibition studies will help us develop a more informative system to safely administer BoNT/A for its medicinal use, thereby improving quality of life.

## 4 Experimental Section

### Media and Reagents of Neural Progenitor Cells (NPCs)

ReN cell VM human neural progenitor cells (NPCs) were purchased from EMD Millipore (Billerica, MA, USA). The cells were plated onto BD Matrigel (BD Biosciences, San Jose, CA, USA)‐coated T25 cell‐culture flasks (BD Biosciences, San Jose, CA, USA) and maintained in DMEM/F12 (Life Technologies, Grand Island, NY, USA) media supplemented with 2 mg heparin (StemCell Technologies, Vancouver, Canada), 2% (v/v) B27 neural supplement (Life Technologies, Grand Island, NY, USA), 20 mg EGF (Sigma–Aldrich, St Louis, MO, USA), 20 mg bFGF (Stemgent, Cambridge, MA, USA), and 1% (v/v) penicillin/streptomycin/amphotericin‐B solution (Lonza, Hopkinton, MA, USA) in a CO_2_ cell culture incubator. Cell‐culture media were changed every 3 d until cells were confluent. For 2D neuron/astrocyte differentiation, the cells were plated onto Matrigel‐coated microfluidic devices with DMEM/F12 differentiation media supplemented with 2 mg heparin, 2% (v/v) B27 neural supplement, and 1% (v/v) penicillin/streptomycin/amphotericin‐B solution without growth factors. Differentiation media was changed every 3–4 days.

### Astrocyte Isolation

After the NPCs had fully differentiated (3 weeks), the cells were detached by accutase 3 min. the cells were collected and centrifuged at regular spin speed. The plates were coated with Matrigel and the cells were added in the culture medium (DMEM/ F12, 10% FBS, and 1% Penicillin). The cells were incubated until confluent. The cells were detached by using accutase and centrifuged at a spin speed of 300 g for 5 min. The step was repeated two times (S1).

### 4.1 Cell Cultures and Differentiation of NPCs

For 3D cultures, BD Matrigel (BD Biosciences) was mixed with the cells (10 × 10^6^cells ml^−1^). The final cell concentration for the mixture was ≈5 × 10^6^ cells ml^−1^ (1:1, 3D thick culture for recruitment) and 2 × 10^6^ cells ml^−1^ (1:5, 3D thin culture for immunostaining). Then transferred 10 µL of cell mixtures into the microfluidic device using prechilled pipettes. The microfluidic devices were incubated for 1 h at 37 °C, during that 3D gels (100–600 µm) formed and media were changed. The 3D‐plated cells were differentiated for 3 weeks before the treatment of BoNT/A; media was changed every 3–4 days.

### Microfluidic Device Fabrication

Negative photoresists SU‐8 10 and SU‐8 100 (MicroChem, Newton, MA, USA) were sequentially patterned using standard lithography on a 4‐inch (10.16 cm) silicon wafer to create a mold for cell migration channels of 20 µm height and chemokine compartments of 100 µm height. The base and a curing agent were mixed at a 10:1 weight ratio (SYLGARD 184 A/B, Dow corning, Midland, MI, USA), poured onto the SU‐8 mold, and cured for 1 h at 25 °C under vacuum and, subsequently, cured for more than 3 h in an oven at 80 °C. The cured poly dimethyl‐siloxane (PDMS) replica was peeled off the mold and holes were punched for fluid reservoirs. Arrayed holes were also laser‐cut (Zing 24, Epilog Laser, Golden, CO, USA) into a thin PDMS membrane of 250 µm thickness (HT 6240, Bisco Silicones, Elk Grove, IL, USA) and an acrylic plate of 6 mm thickness. The machined membrane and plate were glued together using uncured PDMS and incubated at 80 °C overnight. This assembly was irreversibly bonded, first to the PDMS replica using oxygen plasma at 50 mW, 5 cm, for 30 s (PX‐250, March Plasma Systems, Petersburg, FL, USA), and then to a glass bottomed Uni‐Well plate (MGB001‐1‐2‐LG, Matrical Bioscience, Spokane, WA, USA). Immediately after the bonding, 10 µL of BD Matrigel (BD Biosciences, San Jose, CA, USA) were injected into each platform, and it was incubated for 30 min at 37 °C to promote cellular adhesion. The BD Matrigel treated surface was rinsed with autoclaved and 0.2‐µ m filtered water (AM9920, Life Technologies, Grand Island, NY, USA).

### BoNT/A

All the forms of BoNT A, (C, T, DRBoNT, and NAPs were provided by Singh Lab at INADS, N. Dartmouth, MA). Recombinant DRBoNT was formed by adding DRBoNT and NAP's 1:1 Molar ratio and incubated at RT on a rocker for 1 hr. Botulinum neurotoxin, type A was from gram‐positive Clostridium botulinum, which was not expected to have any endotoxins.

### Treatment Condition

To create an environment consistent with the therapeutic dose of administered BoNT/A, the cells were exposed to the toxin with different exposure and recovery times while maintaining the cell viability >75%. For optimizing the treatment conditions, NPCs were treated with BoNT/A 1 nm isolated toxin (T) with different exposure and treatment times. The exposure time varied from 5, 10, and 15 min. and a recovery time of 2, 4, and 6 days. The conditioned media was collected after the recovery phase and a 2^nd^ treatment was given. This time‐dependent treatment cycle was repeated seven times, creating a relevant model similar to therapeutic/drug administration, conditions dependent on cell viability.

### Endopeptidase Activity of BoNT/A

The proteolytic activity of BoNT/A was calculated by measuring the cleavage of SNAP‐25. After the completion of the BoNT/A treatment, media in the culture dishes were removed and washed with ice‐cold PBS. Ice‐cold PBS was removed, and ice‐cold lysis buffer was added to the dishes for 30 min at 4 °C. The cells were collected in the microcentrifuge tube and centrifuged at 16 000 g for 20 min at 4 °C. The supernatant was collected, and the pellet was discarded. SDS‐PAGE running buffer was added to the supernatant, followed by boiling the sample for 3 min in a water bath. The reaction results were examined and viewed by running the precast mini SDS‐PAGE (4‐20% Tris.HCl, 10 wells, Bio‐Rad Laboratories) and Coomassie blue staining. The densitometric analysis of the inhibitor was performed using the Bio‐Rad Image Lab 5.2.1 software. The results reported were representative of three replicates.

### Calcium Imaging

To access the functional synapses and neural connectivity cellular calcium dynamics was monitored. Cellular calcium dynamics were monitored using Rhod‐2 (Life Technologies), a Ca^2+^ indicator (2 mm stock solution in DMSO, 2um in differentiation media). The Oregon Green BAPTA‐1 AM (OGB, Life technologies, USA) was dissolved in dimethyl sulfoxide (DMSO) to make a 1 mm solution. Pluronic F‐127 (P‐6867, Life technologies, USA) was dissolved in DMSO to make a 20% (w/v) solution. Both solutions were mixed at the same volume, followed by adding differentiation media to make 10 µm of OGB. The sample was treated with OGB solution and incubated at 37 °C for 30 min. Finally, cells were washed with fresh neurobasal media and stimulated by increasing the extracellular 56 mm concentration of KCl. For the control, the cells were treated with a buffer containing 2 mm KCl. Fluorescence intensity dynamics were measured using time‐lapse imaging (Texas red filter cube; 20 × objective; Nikon microscope) at 30‐ms framerates. Raw intensity values were extracted using the Nikon software suite, and relative changes in fluorescence intensity to baseline ((*F* – *F*0)/*F*0) were recorded as neuronal calcium signals.

### Complement Activation Peptide (C3a and C5a) Measurements

Complement C3a/des‐Arginated C3a (C3a‐desArg) and C5a/desarginated C5a (C5a‐desArg) were assessed by sandwich ELISA, as described in the protocol provided by the supplier of the kit (BD Pharmingen, city, state), using specific capture antibodies for C3a and C5a and their des‐Arg fragments, respectively. In brief, plates were precoated in human anti‐mouse C3a or C5a capture antibody, diluted in coating buffer, and incubated overnight at 4 °C. Plates were then blocked and incubated with samples and standards for 2 h, followed by a rat anti‐mouse C3a or C5a biotin‐conjugated detection antibody. The amount of captured C3a or C5a was detected using streptavidin‐HRP (BD Pharmingen) and tetramethylbenzidine as a substrate (Sigma–Aldrich), and the plates were read at 450 nm.

### Microglia Preparation

The immortalized human microglia SV40 cell line, derived from primary human microglia, was purchased from Applied Biological Materials, Inc. (ABM, Inc.) and cultured in Prigrow III medium supplemented with 10% (vol/vol) FBS and 1% penicillin/streptomycin in type I collagen‐coated T25‐flasks (ABM, Inc.). Before the experiment, cells were washed using a medium without serum and the cell membrane was labeled with red fluorescent dye (PKH26PCL, Sigma–Aldrich). Briefly, after centrifugation (400 *g* for 5 min), the cells were resuspended in 1 ml of Diluent C (G8278, Sigma–Aldrich) and immediately mixed with 4 µL of dye solution (PKH26PCL, Sigma–Aldrich). The cell/dye mixture was incubated at room temperature for 4 min and periodically mixed by pipetting to achieve a bright, uniform, and reproducible labeling. After incubation, staining was stopped by adding an equal volume (1 ml) of 1% BSA in PBS and incubating for 1 min to remove excess dye. Unbound dye was washed by centrifugation and suspending cells in culture medium (10^6^ cells ml^−1^). For the triculture study, in the microfluidic device, we injected 10 µL of the cell suspension into each platform and 100 µL of a culturing medium was added into side and central extra wells. The loaded 3D microdevices were then incubated at 37 °C supplied with 5% CO2.for 2 days. The cells were treated with BoNT/A toxin and complex with different treatment times. The condition media (CM) was collected after each treatment. The cells were then washed twice with a 10 min time interval with 1% PBS. The washing buffer was replaced with media and incubated at 37 °C for recovery before the next treatment (2,4, and 6 days).

### ELISA: Cytokine Assessment

The collected conditioned media (CM) samples (Microglia preparation section) were tested for TNFα (Thermo Fisher Scientific, KHC3011), IL1β, IL 6 (Thermo Fisher Scientific, BMS213HS) and IFNγ were assessed using their respective ELISA kits, following the manufacturer's instructions. A Synergy 2 ELISA plate reader (BioTek, Winooski, VT, USA) was used to quantify ELISA signals. according to the manufacturer's instructions.

### Cytokine Array

For the simultaneous determination of the relative levels of selected human cytokines and chemokines (MIF, IP10, and IL8), the collected CM from the different samples were mixed with the cocktail of biotinylated detection antibodies from the human cytokine array kit: Proteome profiler array by R&D systems and a bio‐techne brand (#ARY005B). The experiments were performed following the manufacturer's instruction manual.

### Treatment of Microglial Cells

The conditioned media (CM)was collected from the co‐culture after the 5th treatment of T5 and C5 was examined on the single‐cultured microglia. The expression levels of various phenotype markers were monitored on the microglia to investigate the microglial functions in the brain exposed to toxins.

### Migration of Microglial Cells

To test the response of the microglial cells to the soluble cues, microglial cells (20 000 cells mL^−1^) were added in the annular chamber and the conditioned media after the 5th treatment (T5/C5) in the central chamber. The migration of the activated microglial cells to the central chamber for up to 9 days were observed using time‐lapse imaging microscopy.

### Immunostaining

For immunofluorescent stains, the cells and plates and 3D cultures twice were rinsed with PBS (phosphate‐buffered saline). Cells were then fixed at RT, 20 min incubation in fresh 4% paraformaldehyde aqueous solution (157‐4, Electron Microscopy Sciences) followed by rinsing twice with PBS. Cells were permeabilized through incubation in 0.1% Triton X‐100 in PBST (phosphate‐buffered saline with 0.1% Tween 20) for 15 min at RT. Cell‐specific binding was blocked through overnight incubation in 3% human serum albumin in PBST at 4 °C. After 24‐h incubation with the primary antibody solutions at 4 °C, the cells were washed five times. The antibodies and dilutions used are listed in **Table** **4**.

**Table 4:** Antibodies used in the study.

Antibodies	Company	Catalog #	Dilution ratio
pTau (AT8)	Thermo Scientific	MN1020	1:100
GFAP	Sigma–Aldrich	AB5541	1:200
MAP2	Cell Signaling Technology	4542	1:200
NeuN	Abcam	Ab177487	1:100
Cd86	Abcam	AB196564	1:100
CD206	Fisher Scientific	NB6001415	1:100
CD33	Stem cell	60096	1:200
C3aR	Santacruz	sc‐133172 AF647	1:200
C5aR	Biolegend	344306	1:200
TREM2	R&D systems	AF1828	1:100
Synapsin	Abcam	Ab254349	1:100
ChAT	Abcam	ab223346	1:100

### Time‐Lapse Imaging

After microglia loading, cells were recorded using time‐lapse imaging using a fully automated Nikon TiE microscope with a heated incubator to 37 °C and 5% CO2 (10× magnification; Micro Device Instruments, Avon, MA, USA). To achieve accurate cell tracking, the maximum time resolution of the acquisition was 1 frame per s.

### Trainable Weka Segmentation

Trainable Weka Segmentation could provide unsupervised segmentation learning schemes (clustering) and could be customized to employ user‐designed image features or classifiers by combining toolkit Fiji with data mining and machine learning toolkit Waikato Environment for Knowledge Analysis (WEKA) (Arganda‐Carreras et al., 2017). Neuronal and astrocyte populations in culture were differentiated on a cell‐based basis. TWS transforms the segmentation problem into a pixel classification problem in which each pixel could be classified as belonging to a specific segment or class. Specifically, the classifier was trained by the length and direction of neurite extension and was used as the training set for the selected classifier for detecting neurons. Once the classifier was trained, it was used to classify the rest of the input pixels or completely new image data.

## Conflict of Interest

The authors declare no conflict of interest.

## Author Contributions

G.A. hypothesized, designed, and performed all the experiments, analyzed the data, interpreted the results, performed the statistical analysis, and wrote the manuscript. H.C. conceived and oversaw the project, provided guidance, interpreted results, and wrote the manuscript. Y.J.K. helped with the paper's formatting and editing. K.V.D. performed the experiment for Figure S6 (Supporting Information). C.L. helped in providing access to the lab facility facilitating BoNT/A work and helped in writing the manuscript. B.R.S. provided guidance, interpreted the result, and wrote the manuscript.

## Supporting information



Supporting Information

## Data Availability

Data sharing is not applicable to this article as no new data were created or analyzed in this study.
