# Supplementary material for: Botulinum Neurotoxin Induces Neurotoxic Microglia Mediated by Exogenous Inflammatory Responses
Source: Adv Sci (Weinh). 2024 Feb 11;11(15):2305326. doi: 10.1002/advs.202305326 (PMC11022717; doi:10.1002/advs.202305326)
Supplement: Supplementary file 1 — Supporting Information [file ADVS-11-2305326-s001.pdf]

ADVANCED SCIENCE
Open Access

Supporting Information

for *Adv. Sci.*, DOI 10.1002/advs.202305326

Botulinum Neurotoxin Induces Neurotoxic Microglia Mediated by Exogenous Inflammatory Responses

*Ghuncha Ambrin, You Jung Kang, Khanh Van Do, Charles Lee, Bal Ram Singh and Hansang Cho**

Botulinum Neurotoxin Induces Neurotoxic Microglia Mediated by Exogenous Inflammatory Responses.

Ghuncha Ambrin[1,2], You Jung Kang[3,4], Khanh Van Do[3,6], Charles Lee[2], Bal Ram Singh[5], Hansang Cho[3,4,6*]

1. School of Medicine, University of California San Diego, CA 92093, USA
2. Department of Mechanical Engineering and Engineering Sciences, University of North Carolina, Charlotte, NC 28223, USA
3. Institute Quantum Biophysics, Sungkyunkwan University, 2066 Seobu-ro, Jangan-gu, Suwon, Gyeonggi 16419, Republic of Korea
4. Department of Biophysics, Sungkyunkwan University, 2066 Seobu-ro, Jangan-gu, Suwon, Gyeonggi 16419, Republic of Korea
5. Botulinum Research Center, Institute of Advanced Sciences, Dartmouth, MA 02747, USA
6. Department of Intelligent Precision Healthcare Convergence, Sungkyunkwan University, 2066 Seobu-ro, Jangan-gu, Suwon, Gyeonggi 16419, Republic of Korea

***Corresponding Author:**

Hansang Cho, Ph.D., Associate Professor**,** Sungkyunkwan University, 2066 Seobu-ro, Jangan-gu, Suwon, Gyeonggi 16419, Republic of Korea.

Email: h.cho@g.skku.edu

Tel: +82-31-299-4792

Supplementary Figures

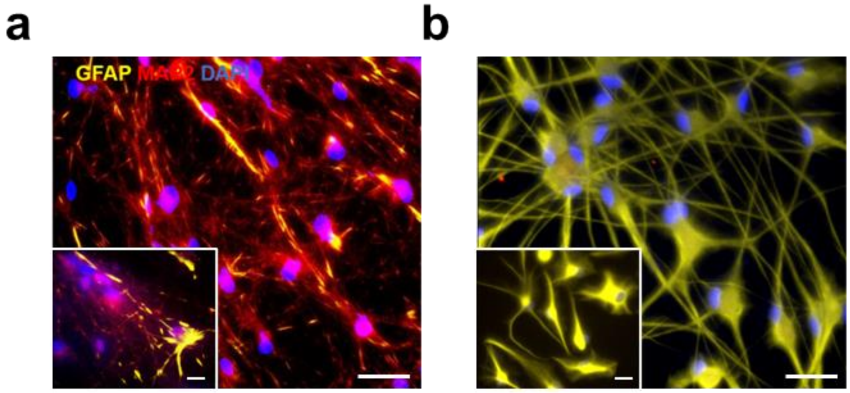

Figure S1. Astrocyte isolation. Astrocyte isolation from the coculture model of differentiated neurons and astrocytes. **a.** Coculture of neurons and astrocytes **b.** Isolated astrocytes. Activated astrocytes stained with GFAP, Neurons with MAP2. Images were taken at 40x and 100x magnification, with scale bars, 50µm, 10µm (insets).

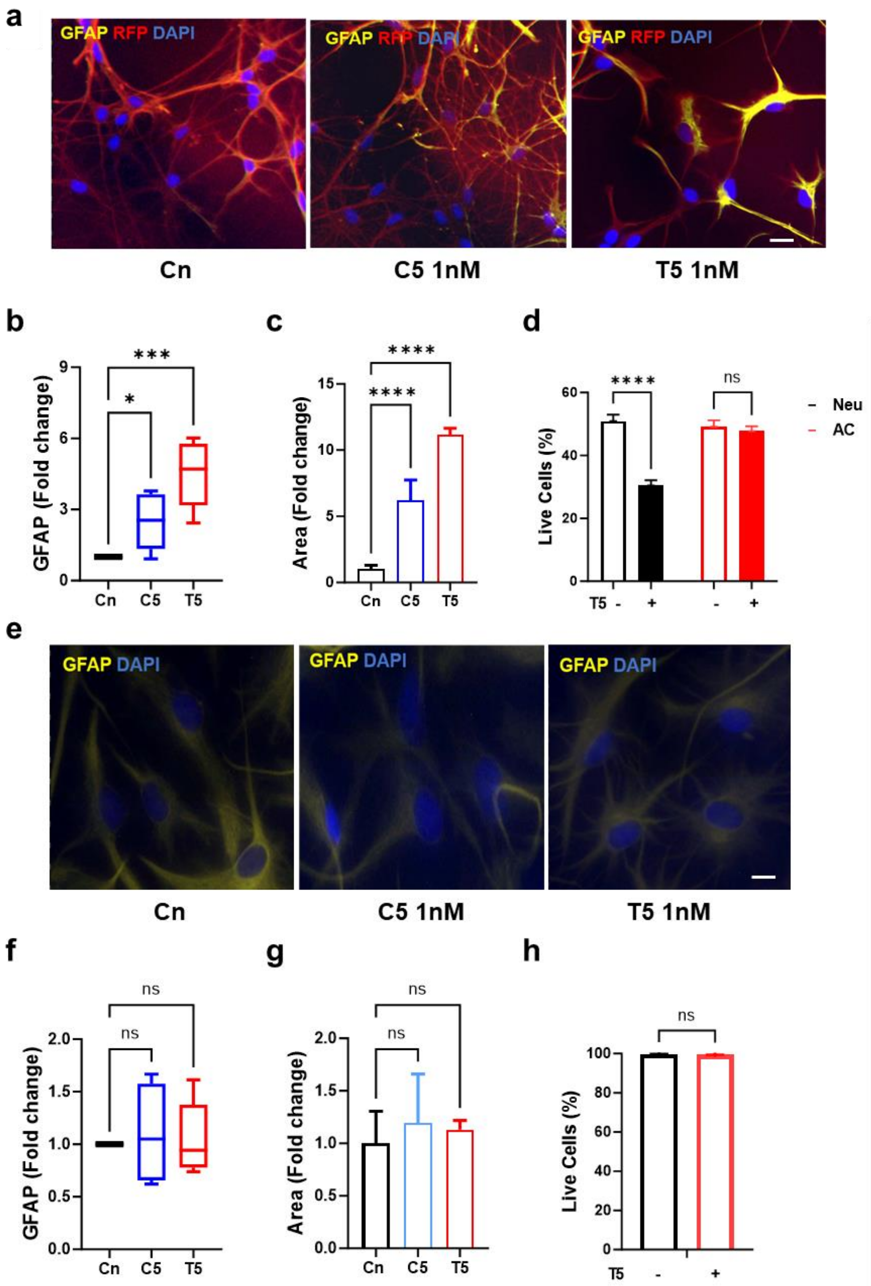

Figure S2. Activation of astrocytes with BoNT/A treatment. **a-c**. A significant increase in the astrocyte activation marker was observed with BoNT/A treatment in the coculture model**. d.** Neuron (Neu) viability was significantly reduced in the coculture system while astrocyte (AC) viability had no significant change**. e-g**. No change in GFAP signaling was observed with C5 and T5 treatment of BoNT/A in the single culture of astrocytes. h. No significant change in AC viability was observed in the single culture model. All experiments were repeated>3 times, all parameters are presented as mean ± s.e.m, ns not significant. All the data was quantified using one-way ANOVA followed by Sidak's multiple comparison test. Scale bars, 10μm

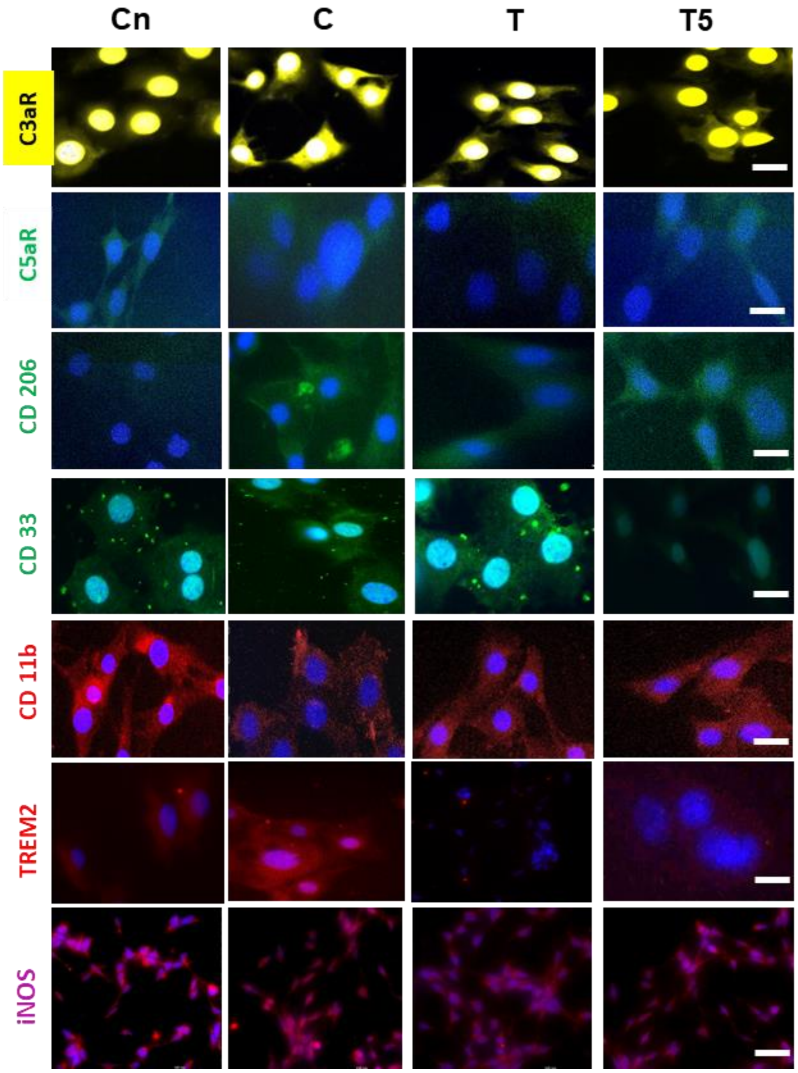

Figure S3. Response of Microglia with BoNT/A treatment. No significant microglia activation was observed with direct T5 and C5 treatment of BoNT/A. The experiment was repeated>3 times in triplicate, scale bars,10μm.

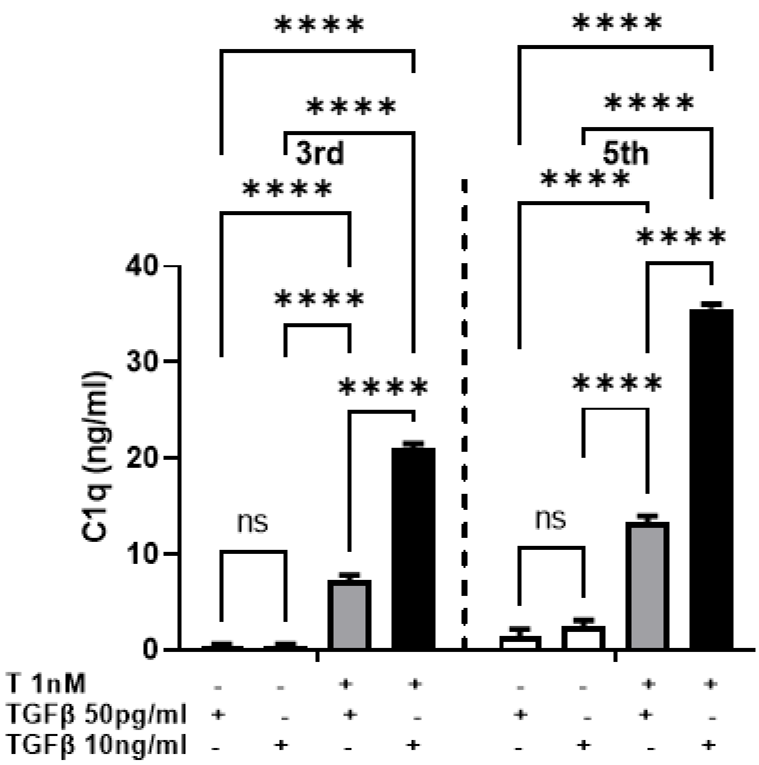

Figure S4. Evaluate C1q secretion with the addition of TGFb. TGFb was added to the system to evaluate the corresponding secretion of C1q at two different concentrations 50pg/ml and 10ng/ml at different treatment intervals. C1q expression increases in the treated model compared to the control where the secretion of C1q seems to be regulated. **** $p<0.0001$. All experiments were repeated ≥ 3 times; all parameters are presented as mean ± s.e.m. p values were calculated using two tailed unpaired t-tests using PRISM.

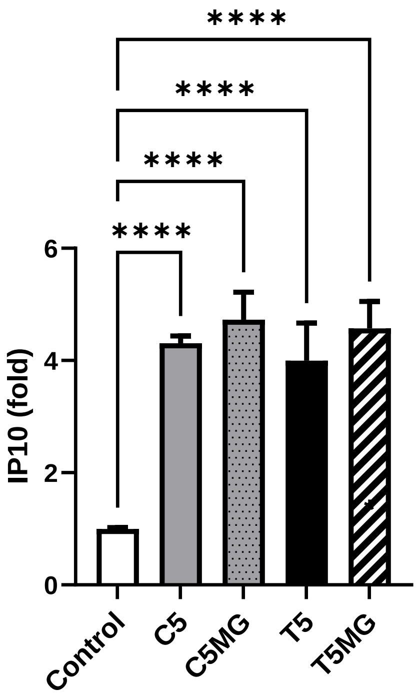

Figure S5. Proinflammatory cytokine secretion of Astrocytes. Astrocytes were the main source of proinflammatory cytokine secretion IP10, increasing ~4-fold with T5 and C5 treatment and showing no significant difference with and without microglial cells. **** p<0.0001. All experiments were repeated ≥ 3 times; all parameters were presented as mean ± s.e.m. p values were calculated using PRISM using two tailed unpaired t-tests.

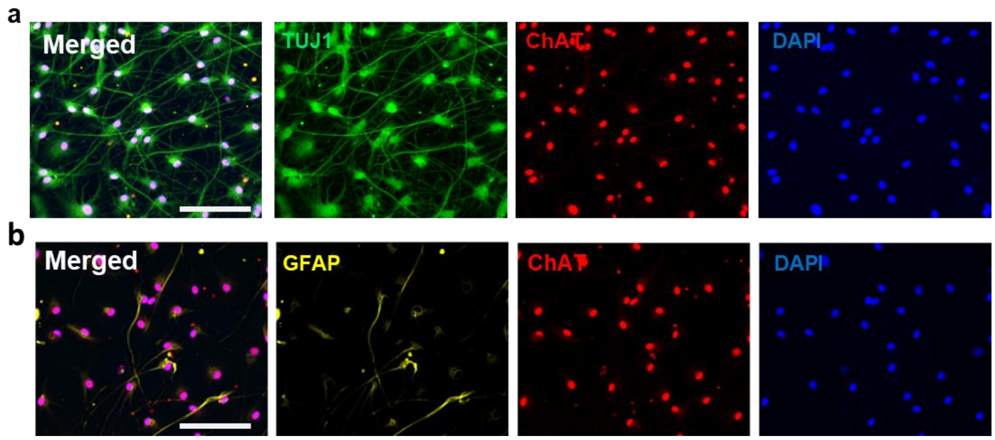

Figure S6. Choline acetyltransferase for the synthesis of acetylcholine. Choline Acetyltransferase ($ChAT^{+}$) is present throughout the mini-brains and expresses the synthesis and release of Ach. The expression level of ChAT (Choline Acetyltransferase) in (**a**) Neurons ($Tuj1^{+}$) and (**b**) Astrocytes ($GFAP^{+}$). Scale bars, 50 μm.

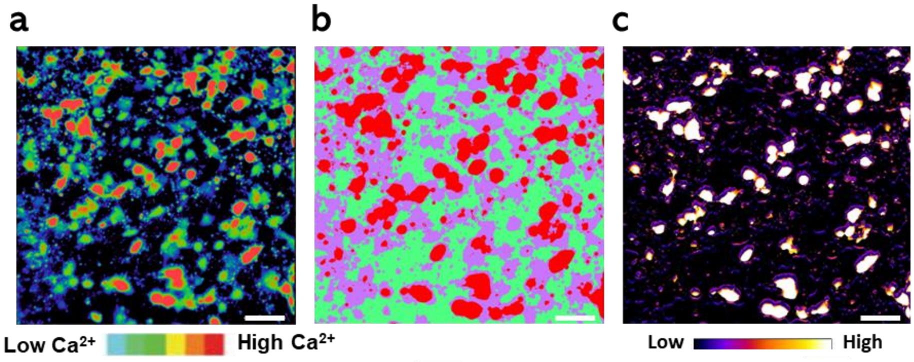

Figure S7. Calcium-mediated neuronal signaling. a. Time-lapse fluorescence microscopy images depicting spontaneous calcium transients**. b.** Probability chart with Weka Segmentation (TWS) identifying neurons and astrocytes. **c**. Calculated image after subtraction of astrocytes, identifying neurons as the cells corresponding to the heat map with calcium transients. Scale bars, 100µm.

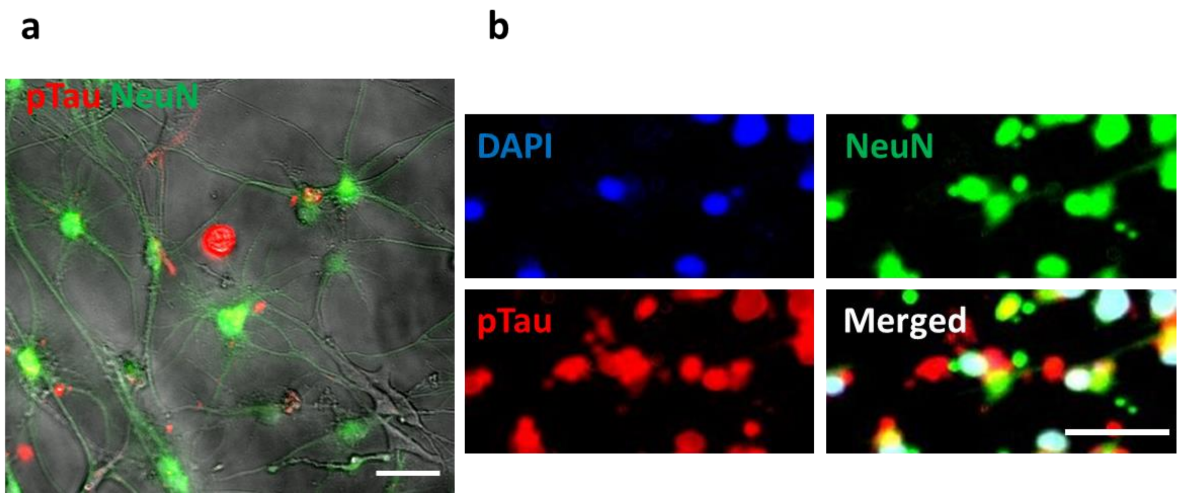

Figure S8. Tau mediated pathogenesis with BoNT/A treatment. **a**. Neuronal damage and pTau aggregates with BoNT/A T5 treatment. **b.** pTau colocalized with neurons (NeuN), scale bars, (a)100 µm, (b) 10µm.a
